# A Rare Case of Acquired Transthoracic Littre's Hernia

**DOI:** 10.1055/s-0039-1696727

**Published:** 2019-10-15

**Authors:** Arthur Curmi, Anthony P. Dimech, Rebecca Dalli, Ayman Mostafa, Joseph Debono

**Affiliations:** 1 Department of Surgery, Mater Dei Hospital, Triq Dun Karm, Msida MSD, Malta

**Keywords:** case report, Meckel's diverticulum, Littre's hernia, diaphragmatic defect, transthoracic, acquired

## Abstract

**Introduction** The Littre hernia is a rare complication of Meckel's diverticulum. Meckel's diverticulum is vestigial remnant of the omphalomesenteric duct occurring in approximately 2% of the general population with an estimated 4 to 16% risk of complications. Usual sites of the Littre hernia include inguinal (50%), umbilical (20%), and femoral (20%). We report a case of an acquired transthoracic Littre's hernia occurring through the left part of the diaphragm triggered by a history of traumatic rib fractures associated with alcohol abuse.

**Case Report** A 71-year-old man presented with 4-day history of worsening shortness of breath, colicky lower abdominal pain, and inability to open bowels despite passing flatus, without nausea or vomiting. His past medical history was remarkable for multiple traumatic rib fractures caused by falls which were associated with excessive alcohol consumption. A noncontrast computed tomography (CT) scan of the abdomen and pelvis showed distended jejunal loops containing air/fluid levels likely resulting from herniated jejunum between the left chest wall and left diaphragm. An urgent laparotomy was performed which revealed small bowel and omentum herniating through a small defect in the left posterior hemidiaphragm. The contents of the sac were reduced and a Meckel's diverticulum was found inside the sac, characteristic of Littre's hernia. The diaphragmatic defect was closed and the Meckel diverticulum stapled and excised.

**Discussion** Herniation of Meckel's diverticulum through the diaphragm most commonly occurs in the pediatric population. Acquired transthoracic Littre's hernia is rare and may arise following thoracobdominal trauma caused by surgery, motor vehicle accidents, and falls from height. Left-diaphragmatic tears are characteristically more clinically apparent and symptomatic than the right since the liver often has a protective effect on the right part of the diaphragm. Herniation of abdominal contents in the chest cavity causes respiratory distress and requires urgent surgical correction. Diagnosis is often delayed since diaphragmatic hernia tends to present very late after the initial trauma, subjecting the patient to possible life-threatening complications. While it is easier to reduce the herniated contents and repair the diaphragm via a thoracic approach, laparotomy is often preferred in cases of acute trauma associated with intra-abdominal injuries. Repair of Littre's hernia then consists of resection of the diverticulum and herniorraphy.

**Conclusion** Internal Littre's hernia is usually of congenital origin. This is the first case of a transthoracic Littre's hernia caused by traumatic rib fractures. Hence, it is of utter importance that a clinician is aware of such uncommon pathology.

Meckel's diverticulum is the most common congenital malformation of the gastrointestinal tract and is a vestigial remnant of the omphalomesenteric duct. During fetal development in-utero, this structure connects the primitive gut with the yolk sac of the embryo and usually regresses between the sixth and seventh week of life. It is a true diverticulum involving all three layers of the intestinal wall; however, it may also contain ectopic gastric, pancreatic mucosa, and less commonly duodenal and colonic mucosa. 1

As far as the characteristics of Meckel's are concerned, literature often quotes the rule of two's. The prevalence of Meckel's in the general population is reported to be approximately 2%, although it is difficult to obtain a true estimate since the condition is typically silent. It reaches 2 inches length (5 cm) and occurs within 2 feet (60 cm) proximal to the ileoceacal valve on the antimesenteric border of the ileum. 2 It is twice more common in males and, if symptoms do occur, they usually present with painless rectal bleeding in the first decade of life with an average age of 2 years. The rectal bleeding is described as currant jelly and is due to ulceration caused by acid secretion by ectopic gastric mucosa. 3

Littre's hernia is the protrusion of Meckel's through a potential abdominal opening. Two kinds are recognized as follows: (1) a combined Littre's hernia (when a Meckel's diverticulum is accompanied by another viscus in the hernia sac) and (2) true Littre's hernia (a solitary Meckel's diverticulum). 4 We report a case of an acquired transthoracic Littre's hernia occurring through the left part of the diaphragm triggered by a history of traumatic rib fractures associated with alcohol abuse.

## Case Report

A 71-year-old man presented to the emergency department at Mater Dei Hospital (Malta) with 4-day history of colicky lower abdominal pain, without nausea or vomiting, and an inability to open bowels despite passing flatus. He also complained of a cough productive of greenish sputum and worsening shortness of breath. On examination, he was afebrile with a blood pressure of 179/80 mm Hg, pulse of 90 beats per minute, and peripheral oxygen saturation of 92% on room air. There were bilateral fine chest crepitations on auscultation. He had lower abdominal tenderness on deep palpation but no signs of peritonism. Bowel sounds were sluggish and no hernias were identified clinically.

Urgent laboratory investigations revealed a normal white blood cell count (4.52  × 109/L), microcytic anemia (hemoglobin, 13.1 g/dL; mean cell volume, 94.4 fL), acute kidney injury (urea, 25.8 mmol/L; creatinine, 225 µmol/L; estimated glomerular filtration rate (eGFR), 30 mL/min/1.73m ^2^ ), and serum potassium of 5.65 mmol/L. His international normalized ratio (INR) was 2.71, C-reactive protein 88.6 mmol/L, and lactate 0.4 mmol/L. A chest X-ray identified a chronic left sided pleural effusion with fibrotic changes in the right lower lung zone. Evidence of a healed left seventh rib fracture and a large hiatus hernia were also present. On abdominal X-ray, multiple dilated loops of small bowel were noted.

His past medical history was remarkable for hypertension, congestive cardiac failure, cerebrovascular accident, right subclavian vein thrombosis, left carotid endarterectomy, right pulmonary empyema, left sided pneumothorax with surgical emphysema following fifth and sixth rib fractures in 2011 (requiring chest drain), and traumatic left 6 to 12th rib fractures in 2014. He was also an exsmoker and exhibited alcohol misuse. His medications included warfarin (dose adjusted according to INR), folic acid 5 mg daily, lactulose 15 mL daily, simvastatin 10-mg nocte, and bumetanide 0.5 mg daily.

**Fig. 1:**
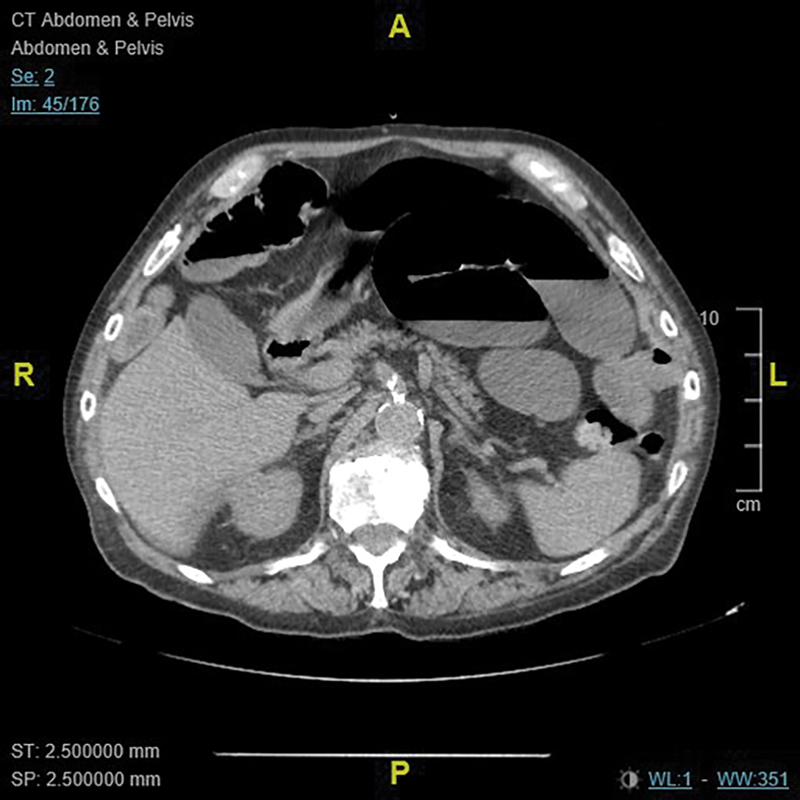
Distended jejunal loops containing air/fluid levels consistent with small bowel obstruction.

**Fig. 2:**
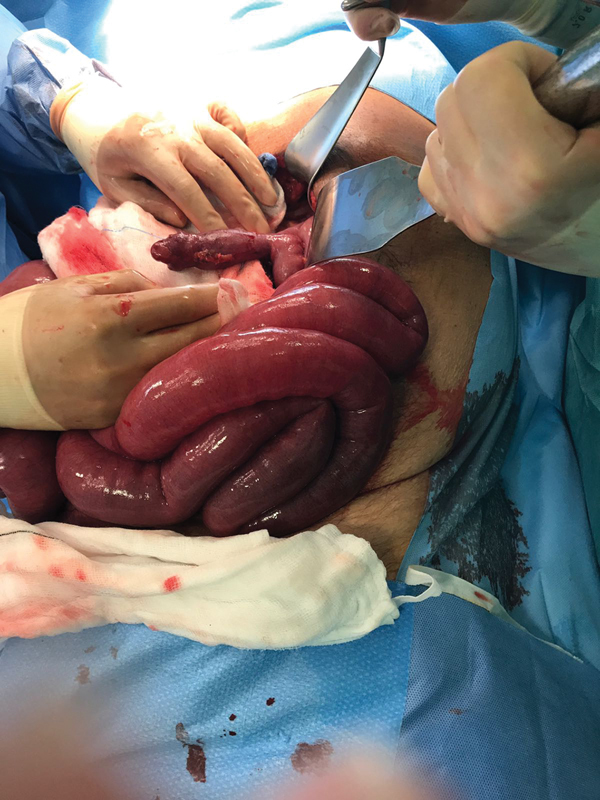
Intraoperative image of Meckel's diverticulum on the antimesenteric border of the ileum.

A noncontrast computed tomography (CT) scan of the abdomen and pelvis showed distended jejunal loops containing air/fluid levels likely resulting from herniated jejunum between the left chest wall and left diaphragm ( Fig. 1 ). No obvious masses or large bowel pathology were noted. A combination of coamoxiclav and clarithromycin (doses titrated according to renal function) were started for his respiratory infection. He was subsequently consented for an urgent exploratory laparotomy for possible subacute small intestinal obstruction secondary to a diaphragmatic hernia. During laparotomy, small bowel and omentum were seen herniating through a small defect in the left posterior hemidiaphragm. The contents of the sac were reduced and a Meckel's diverticulum was found inside the sac, characteristic of a Littre's hernia ( Fig. 2 ). This was later confirmed on histology and was found to be 55 mm in length and 35 mm in diameter without malignant features or ectopic elements. The diaphragmatic defect was closed with nonabsorbable polypropylene 0 sutures and the Meckel's diverticulum stapled and excised. The small bowel was milked retrogradely and its contents emptied through a nasogastric tube. A nonsuction drain was placed in the left paracolic gutter.

Postoperatively, the patient made a steady recovery. Regular chest physiotherapy was performed and thromboembolic deterrent (TED) stockings applied. The drain was removed 4 days later and antibiotics stopped after 9 days. He was deemed fit for discharge home 10 days into his admission.

## Discussion

Meckel's diverticulum is often an incidental finding during laparoscopy or laparotomy performed for nonrelated reasons. According to Johann Friedrich Meckel himself, the incidence of complications associated with Meckel's is 25%, although risk of complications in recent research ranges from 4 to 16%. Complications tend to occur more in males and decrease with age with the majority of them occurring in the pediatric population. Common presentations for a symptomatic Meckel's are intestinal obstruction, gastrointestinal hemorrhage, and diverticulitis with or without perforation. 5

Littre's hernia is an unusual complication of Meckel's diverticulum. Usual sites of Littre's hernia include inguinal (50%), umbilical (20%), and femoral (20%). 6 In this particular case, the hernia occurred through a defect in the left part of the diaphragm. The cause was a history of traumatic rib fractures; our patient sustained fifth and sixth rib fractures associated with a surgical emphysema and a left sided pneumothorax that required chest drain in 2011, and left 6—12th rib fractures 3 years later. In both cases, the fractures were due to falls associated with alcohol consumption.

Meckel's diverticulum has herniated through the diaphragm in other reported cases, but these were of congenital origin. 7 8 We could only find one other case in literature of a Littre's hernia occurring through an acquired diaphragmatic defect. This followed minimally invasive esophagectomy for curative management of esophageal cancer 6 years before. 9

The incidence of acquired diaphragmatic hernia following trauma ranges from 0.8 to 1.6%. 10 Traumatic diaphragmatic defects usually result from penetrating injuries or blunt thoracoabdominal trauma often associated with motor vehicle accidents or falls from height. Left-diaphragmatic tears (80%) are characteristically more clinically apparent and symptomatic than the right (15%) since the liver often has a protective effect on the right part of the diaphragm. The left part is relatively unprotected and, therefore, represents an area of weakness. The right diaphragm is also stronger than the left which is in itself weakened by a line of embryonic fusion between the lumbar and costal parts. 11 In 5% of the cases, the defect is bilateral. Although penetrating diaphragmatic defects due to rib fractures are initially very small and unremarkable, the defect will generally increase in size over time as the negative intrathoracic pressure generated by inspiration causes herniation of abdominal viscera into the chest. A positive pressure gradient exists between the pleural and peritoneal space. This greatly facilitates herniation of abdominal contents through left diaphragmatic defects. When the diaphragmatic tear becomes very large and associated with herniation of abdominal organs, the patient usually presents to hospital with severe respiratory compromise, generalized chest and abdominal pain, nausea, decreased breaths on the side of the diaphragmatic defect, and auscultation of bowel sounds in the chest. 12 Since omentum is pliable and mobile, it is often the first abdominal structure to herniate into the thorax.

A particular challenge in the successful treatment of acquired diaphragmatic hernia is early diagnosis which can sometimes be excessively delayed, especially in cases when the degree of diaphragmatic defect has not been established in the acute period of trauma. It is relatively common for a diaphragmatic hernia to present very late after the initial trauma with an increased risk of life-threatening complications.

Herniation of abdominal contents in the chest cavity causes respiratory distress and requires urgent surgical correction. Laparotomy is the most common approach in cases of acute trauma associated with intra-abdominal injuries. 13 In the absence of abdominal injuries, a thoracotomy is preferred since it is easier to reduce the herniated contents and repair the diaphragm. 14 In chronic phases of trauma, the possibility of adhesions between the herniated abdominal viscera should be considered as this may make an abdominal approach difficult. In several cases, both thoracotomy and laparotomy are combined in a single operation.

Surgery usually involves reduction of hernia contents followed by repair of the diaphragmatic defect. In our case, a midline supraumbilical laparotomy was performed. Repair of Littre's hernia consists of resection of the diverticulum and herniorrhaphy. Pneumothorax and septicemia are the most common complications following surgical intervention and these were absent in our case.

## Conclusion

This case addresses an extremely rare finding both in terms of the presence of Meckel's and in the way it caused complications. As identified in a single previous case report, Meckel's herniation through a diaphragmatic defect in an adult typically occurs as a late complication of surgery. Our report is the first of its kind involving repeated trauma to the lower ribs on the left side. This contrasts with more common forms of internal Littre's hernia which are mostly of congenital nature. While the etiology of small bowel obstruction is diverse, especially with a history of abdominal or pelvic surgery, a clinician must not be caught out by the less common precipitants.
